# Biological Characterization and Clinical Value of OAS Gene Family in Pancreatic Cancer

**DOI:** 10.3389/fonc.2022.884334

**Published:** 2022-06-03

**Authors:** Li-Juan Gao, Jia-Lei Li, Rui-Rui Yang, Zhong-Mei He, Min Yan, Xia Cao, Ji-Min Cao

**Affiliations:** ^1^ Key Laboratory of Cellular Physiology, Ministry of Education, Shanxi Medical University, Taiyuan, China; ^2^ Department of Physiology, Shanxi Medical University, Taiyuan, China

**Keywords:** pancreatic cancer, OAS gene family, prognosis, immune infiltration, biomarker

## Abstract

**Background:**

OAS gene family plays an important role in antiviral process, but its role in pancreatic cancer has not yet been studied.

**Methods:**

We analyzed the expression, prognostic value and biological function of the OAS gene family in human pancreatic cancer through comprehensive bioinformatic analysis and cellular level validation.

**Results:**

OAS family was highly expressed in pancreatic cancer, and this high expression significantly affected the clinical stage and prognosis of the tumor. OAS gene family was closely related to the immune infiltration of pancreatic cancer, especially neutrophils and dendritic cells, and many immune-related factors and pathways are enriched in the tumor, such as type I interferon signaling pathway and NOD-like receptor signaling pathway.

**Conclusion:**

Taken together, high expression of OAS family is closely related to poor prognosis of pancreatic cancer. OAS gene family may serve as the biomarker and even therapeutic target of pancreatic cancer.

## Introduction

Pancreatic cancer is one of the most malignant tumors in the world, bringing a great threat to human health and life. Pancreatic cancer is called as the “king of cancer”, because of its difficulty in early diagnosis, high degree of malignancy, and extremely poor prognosis (1, 2). The five-year survival rate is less than 10% (3), and even surgery is ineffective. When most patients fall sick and seek medical treatment, the cancer has already spread locally or developed multi-organ metastasis and lost optimal treatment time. Thence, early detection and treatment may provide more survival opportunities for patients with pancreatic cancers (4). The occurrence and development of pancreatic cancer is a multi-stage and multi-gene change process. At present, its pathogenesis is still unclear. In recent years, with the rapid development of genetic diagnosis technology, some genes have been found closely related to pancreatic cancer.

The 2’,5’-oligoadenylate synthetase (OAS) gene family has been discovered and characterized as a family of interferon (IFN)-induced enzymes which can convert ATP to 2’,5’-linked oligomers of adenosine in the presence of double-stranded (ds)RNA, then the oligomers activate RNaseL (5, 6). RNaseL is an endonuclease, can degrade all single-stranded RNA and cut 18S or 28S rRNA in cells, thus inhibit the proliferation of RNA virus. Therefore, OAS/RNaseL antiviral system can inhibit the replication of many single-stranded RNA viruses in the cytoplasm. The OAS family consists of four members, including OAS1, OAS2, OAS3, and OASL, they are encoded by distinct genes clustered on the 2’,5’-OAS locus on human chromosome 12 (7, 8). OAS genes are related to many diseases, such as innate immune-activated diseases (9), HIV infection (10), chronic skin disease (11), breast cancer (12), etc. However, the role of OAS family in pancreatic cancer is still unclear and thus deserves extensive studies.

In the present study, we comprehensively analyzed the expression, clinical prognostic value and biological functions of OAS family in human pancreatic cancer using a variety of databases including GTEx, Oncomine, GEPIA, TIMER, OncoLnc, Kaplan-Meier plotter and metascape. We further verified the bioinformatic results in human pancreatic cancer cell lines. The findings illustrated an important role of OAS family in pancreatic cancer and the potential biological mechanism.

## Results

### High mRNA Levels of OAS Gene Family in Pancreatic Cancer Tissues

**Figure 1:**
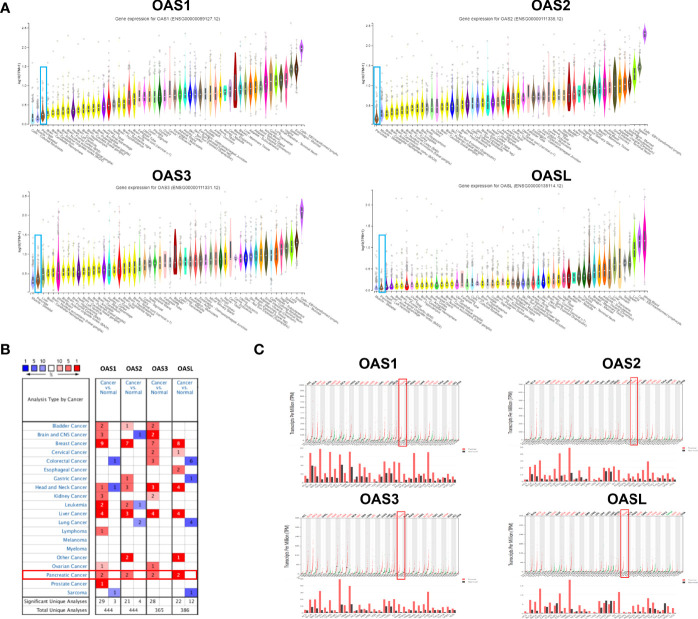
The mRNA expression patterns of OAS1, OAS2, OAS3, and OASL in various normal tissues of the human body and different types of human cancer. **(A)** Ranks of the expression levels of OAS gene family in various human normal tissues from GTEx database. OAS family expression in pancreatic cancer was ranked as a very low expression. **(B)** Results of Oncomine database analysis, each gene had two evidences to prove its high expression in pancreatic cancer (fold change > 2, P < 0.0001). **(C)** Results of GEPIA database analysis which proved high expression of OAS family in pancreatic cancer. Black fonts, red fonts and green fonts on the top of each subpanel indicate no change, high expression and low expression, respectively).

We searched the mRNA levels of OAS family in normal pancreatic tissues in GTEx database, and checked the mRNA levels in pancreatic cancer tissues in Oncomine and GEPIA databases. Results showed that the mRNA levels of OAS family members were very low in normal pancreatic tissues (**Figure 1A**), but were high in pancreatic cancer tissues (**Figure 1B, C**), suggesting that the transcription levels of OAS gene family might be an indicator of pancreatic cancer.

### Differential mRNA Expressions of OAS Gene Family Members in Pancreatic Cancer

We further analyzed the mRNA levels of OAS1, OAS2, OAS3, and OASL in pancreatic cancer tissues and normal pancreatic tissues based on Oncomine and GEPIA databases. Analysis on the Oncomine database showed that all the four OAS members were significantly upregulated in pancreatic cancer tissues compared with the normal pancreatic tissues. The analysis was performed in six sub-datasets, with |logFC| > 1.5 (fold change, FC) and P < 0.05, including Pei Pancreas (13), Badea Pancreas (14), Logsdon Pancreas (15), Segara Pancreas (16), Iacobuzio-Donahue Pancreas 2 (17), and Grutzmann Pancreas (18) (**Table 1**).

**Table 1:** The mRNA levels of OAS family in different types of pancreatic cancer and normal pancreatic tissues at transcriptome level (ONCOMINE).

Gene	Types of Bladder Cancer vs. Normal	Fold Change	P-value	t-Test	References
OAS1	Pancreatic Ductal Adenocarcinoma (39) vs. Normal (39)	2.583	2.76E-8	6.056	Badea Pancreas
	Pancreatic Carcinoma (36) vs. Normal (16)	5.442	1.67E-7	6.778	Pei Pancreas
	Pancreatic Carcinoma (11) vs. Normal (6)	2.241	5.06E-4	4.145	Segara Pancreas
	Pancreatic Adenocarcinoma (10) vs. Normal (5)	3.173	1.47E-4	4.916	Logsdon Pancreas
	Pancreatic Adenocarcinoma (12) vs. Normal (3)	5.530	0.004	4.073	Iacobuzio-Donahue Pancreas 2
OAS2	Pancreatic Ductal Adenocarcinoma (39) vs. Normal (39)	2.721	1.09E-8	6.352	Badea Pancreas
	Pancreatic Ductal Adenocarcinoma (11) vs. Normal (11)	1.961	0.008	2.620	Grutzmann Pancreas
	Pancreatic Carcinoma (36) vs. Normal (16)	2.129	1.02E-6	5.406	Pei Pancreas
	Pancreatic Carcinoma (11) vs. Normal (6)	1.995	0.007	2.909	Segara Pancreas
	Pancreatic Adenocarcinoma (12) vs. Normal (5)	3.563	2.06E-4	4.514	Iacobuzio-Donahue Pancreas 2
OAS3	Pancreatic Ductal Adenocarcinoma (39) vs. Normal (39)	2.540	1.23E-10	7.360	Badea Pancreas
	Pancreatic Carcinoma (36) vs. Normal (16)	3.562	2.04E-7	6.130	Pei Pancreas
OASL	Pancreatic Ductal Adenocarcinoma (39) vs. Normal (39)	1.820	8.85E-7	5.189	Badea Pancreas
	Pancreatic Carcinoma (36) vs. Normal (16)	6.317	8.34E-13	9.437	Pei Pancreas
	Pancreatic Adenocarcinoma (10) vs. Normal (5)	77.098	1.37E-5	7.786	Logsdon Pancreas

Datasets of Badea Pancreas, Pei Pancreas, Segara Pancreas, Logsdon Pancreas and Iacobuzio-Donahue Pancreas 2 were used to examine the mRNA levels of OAS1. In Badea Pancreas dataset, OAS1 was highly expressed in pancreatic ductal adenocarcinoma with a fold change of 2.583 compared with normal pancreatic tissues. In Pei Pancreas and Segara Pancreas datasets, OAS1 was overexpressed with a fold change respectively of 5.442 and 2.241 in pancreatic carcinoma compared with the normal tissues. In Logsdon Pancreas and Iacobuzio-Donahue Pancreas 2 datasets, OAS1 was 3.173 and 5.530 times higher in pancreatic adenocarcinoma compared with normal tissues (**Table 1**).

Datasets of Badea Pancreas, Grutzmann Pancreas, Pei Pancreas, Segara Pancreas, and Iacobuzio-Donahue Pancreas 2 were employed to check the mRNA levels of OAS2. In Badea Pancreas and Grutzmann Pancreas datasets, OAS2 was overexpressed with a fold change respectively of 2.721 and 1.961 in pancreatic ductal adenocarcinoma compared with the respective normal tissues. In Pei Pancreas and Segara Pancreas datasets, OAS2 was 2.129 and 1.995 times higher in pancreatic carcinoma tissues compared with respective normal pancreatic tissues. In Iacobuzio-Donahue Pancreas 2 dataset, OAS2 was highly expressed in pancreatic adenocarcinoma with a fold change of 3.563 compared with normal pancreatic tissues (**Table 1**).

Datasets of Badea Pancreas and Pei Pancreas were applied to examine the mRNA levels of OAS3. In Badea Pancreas dataset, OAS3 was upregulated with a fold change of 2.540 in pancreatic ductal adenocarcinoma. In Pei Pancreas dataset, OAS3 was 3.562 times higher in pancreatic carcinoma compared to normal pancreatic tissues (**Table 1**).

Datasets of Badea Pancreas, Pei Pancreas and Logsdon Pancreas were used to check the mRNA levels of OASL. In Badea Pancreas dataset, OASL was upregulated with a fold change of 1.820 in pancreatic ductal adenocarcinoma. In Pei Pancreas dataset, OAS3 was 6.317 times higher in pancreatic carcinoma compared to normal tissues, In Logsdon Pancreas dataset, OASL was 77.098 times higher in pancreatic adenocarcinoma compared to the normal pancreatic tissues (**Table 1**).

**Figure 2:**
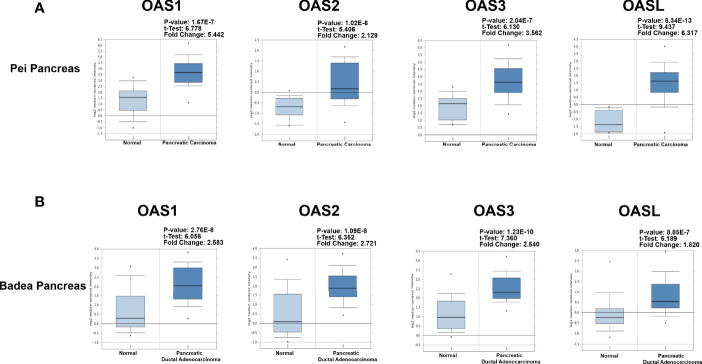
Detailed expression levels of OAS gene family in pancreatic cancer from Oncomine database. **(A)** Box plots of OAS1, OAS2, OAS3, and OASL expression levels in Badea and Pei Pancreas dataset of pancreatic cancer. **(B)** Box plots of OAS1, OAS2, OAS3, and OASL expression levels in Badea Pancreas dataset.

**Figure 3:**
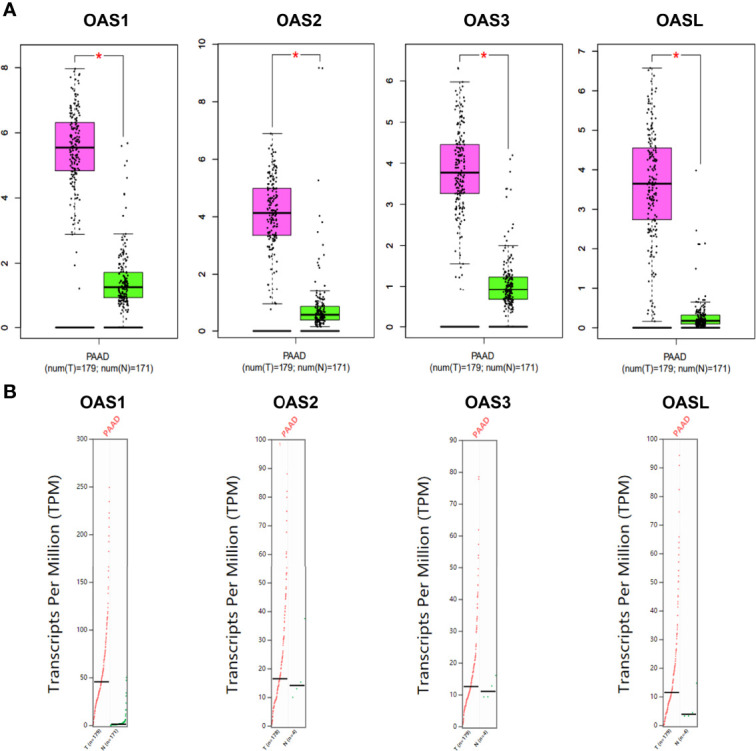
Expression characterization of OAS1, OAS2, OAS3, and OASL in pancreatic cancer derived from GEPIA database. **(A)** Box plots. **(B)** Dot plots. “*” indicates significant difference in the expression levels of OAS genes between tumor and normal tissues.

To better characterize the transcriptional levels of OAS gene family in pancreatic cancer, we selected two datasets, i.e., Badea and Pei Pancreas datasets from Oncomine database, to show the precise mRNA expression of OAS family in pancreatic cancer (**Figure 2A, B**). In the two datasets, the mRNA levels of the four OAS genes were all upregulated in pancreatic cancer compared with normal pancreatic tissues. In addition, by analyzing the GEPIA database, the mRNA levels of OAS1, OAS2, OAS3, and OASL were all significantly higher in pancreatic cancer tissues than normal pancreatic tissues (**Figure 3**).

### Validation of mRNA and Protein Expressions of OAS Family by Quantitative Real-time PCR (qPCR) and Western Blotting in Pancreatic Cancer Cell Lines

**Figure 4:**
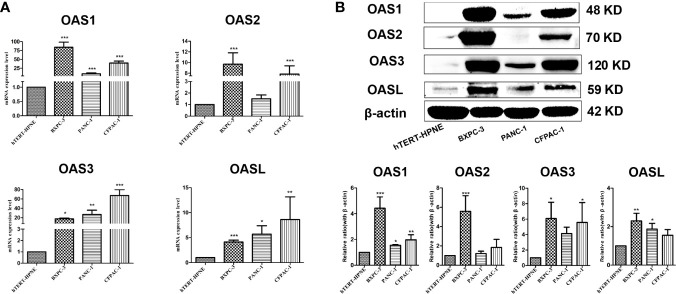
Results of qPCR and western blotting of OAS gene family in pancreatic cancer cell lines (BxPC-3, PANC-1, CFPAC-1) and pancreatic normal cell line (hTERT-HPNE). **(A)** mRNA levels of qPCR. **(B)** Representative electrophoresis bands of western blotting. *P < 0.05, **P < 0.01, ***P < 0.001.

To further verify the above bioinformatic results shown in **Figures 1**, **2**, **3**, and **Table 1**, RT-qPCR and Western blotting were performed in three human pancreatic cancer cell lines (BxPC-3, PANC-1, CFPAC-1) and normal pancreatic cell line (hTERT-HPN). qPCR results showed that the mRNA levels of OAS family in pancreatic cancer cells were higher than normal pancreatic cells (**Figure 4A**), which are consistent with bioinformatic results. Western blotting results showed that the protein expression levels of OAS1, OAS2, OAS3, and OASL were significantly elevated in pancreatic cancer cells compared with normal pancreatic cells (**Figure 4B**).

### Immunohistochemical Features of OAS Protein Family in Human Pancreatic Cancer and Normal Pancreatic Tissues

**Figure 5:**
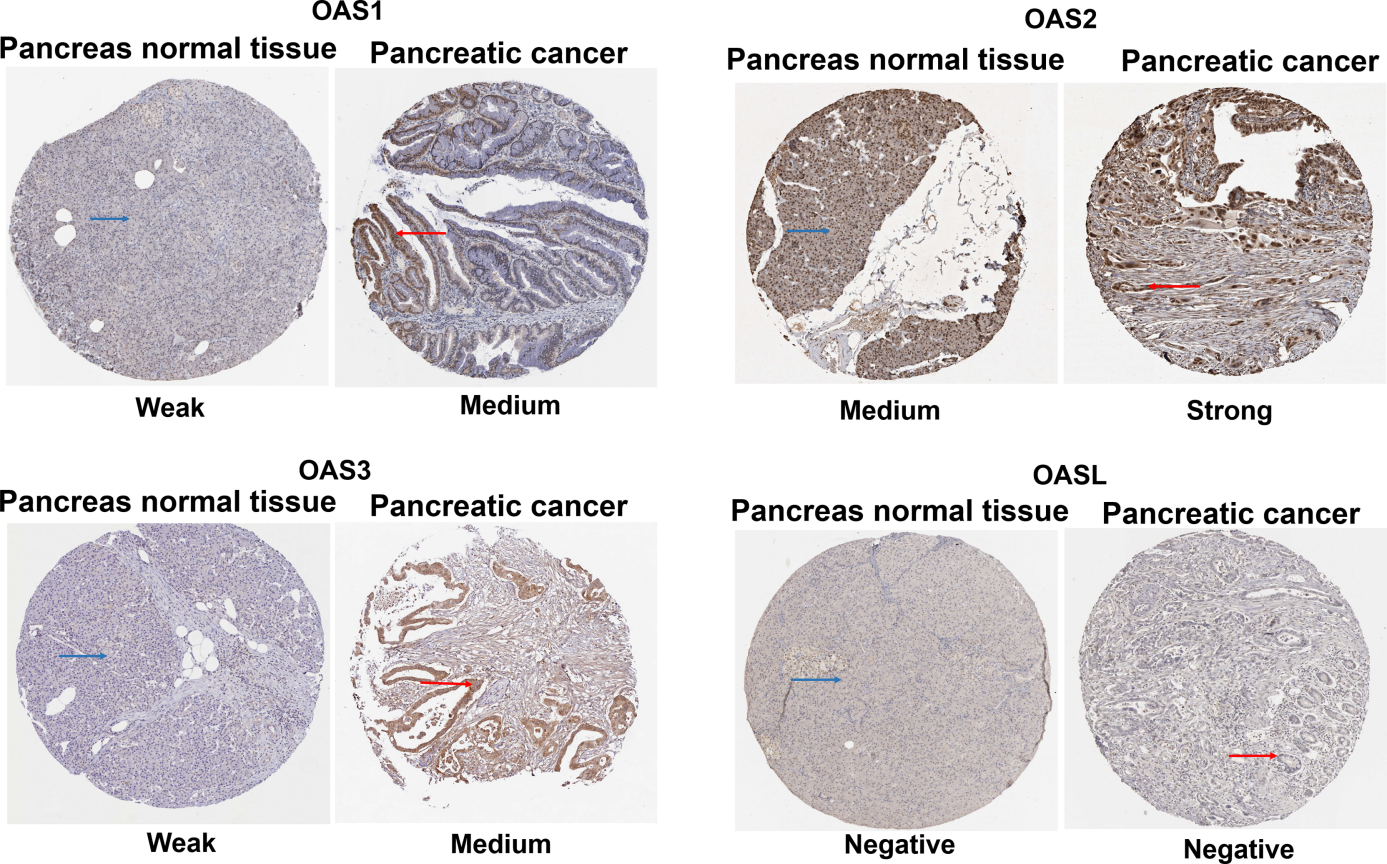
Immunohistochemical stains from the Human Protein Atlas Project showing the representative protein expression of OAS gene family in normal pancreatic tissue and pancreatic cancer.

Immunohistochemical stains of OAS1, OAS2, OAS3 and OASL proteins in human pancreatic cancer tissues and normal pancreatic tissues were searched from the HPA database. Results (**Figure 5**) revealed that the positive OAS1 protein stains were weak in normal pancreatic tissues but showed medial signal intensity in pancreatic cancer tissues. OAS2 stains showed medial intensity in normal pancreatic tissues but showed strong intensity in pancreatic cancer tissues. OAS3 stains exhibited weak intensity in normal pancreatic tissues but showed medial intensity in pancreatic cancer tissues. OASL positive stains were not detected in both pancreatic cancer and normal pancreatic tissues.

### Transcription Factors and microRNAs Regulating OAS Gene Expressions in Pancreatic Cancer

**Figure 6:**
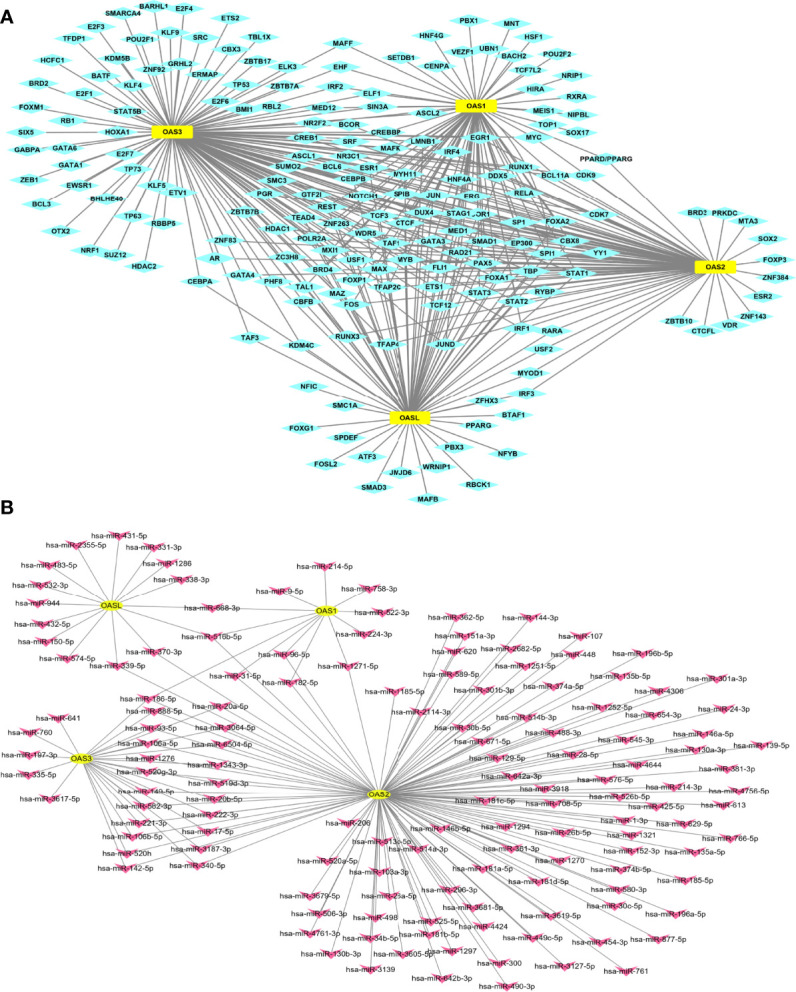
TF-target and miRNAs-target that regulate OAS gene family. **(A)** TF-target result. **(B)** miRNAs-target result.

Gene expression is regulated by many factors including transcription factors (TFs) and microRNAs (miRNAs). We used TF-target database (hTFtarget) and miRNA-target database (Starbase and Targetscan) to further clarify the upstream TFs and miRNAs that regulate the expression of OAS genes. We found 193 TFs (**Figure 6A**) and 140 miRNAs (**Figure 6B**) that regulate the expression of OAS gene family. The numbers of miRNAs that regulate OAS1, OAS2, OAS3, and OASL were respectively 12, 118, 27 and 15, as shown by the intersection selected from Starbase and Targetscan databases (**Supplementary Figure 1**).

### Clinicopathological Parameters and Prognostic Values of OAS Family in Pancreatic Cancer

**Figure 7:**
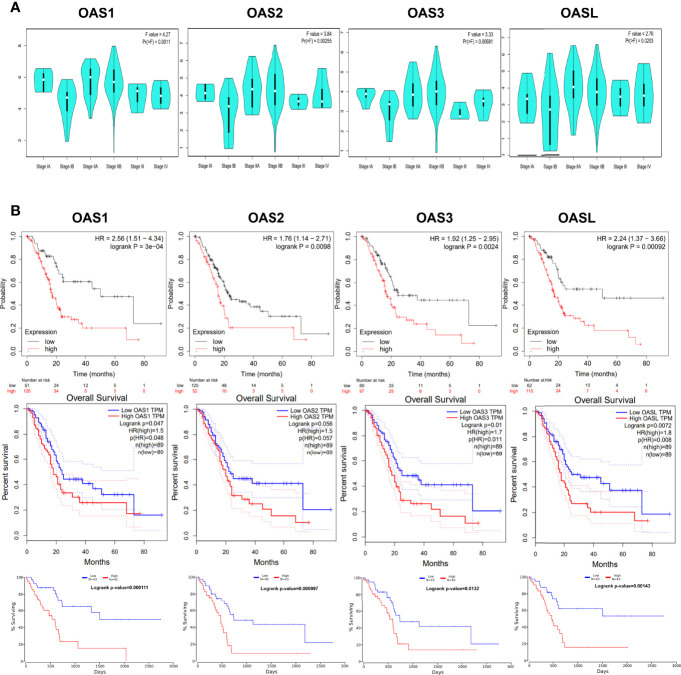
Association of OAS family expression level with clinical stage and prognosis. **(A)** The expression of OAS family was significantly different in each clinical stages of pancreatic cancer. P < 0.05. **(B)** Prognostic value of OAS gene family for pancreatic cancer in Kaplan-Meier (KM) plotter, GEPIA, and OncoLnc database.

Gene expression level may affect cancer development (19). We revealed the relationship between the expression levels of the four OAS family members and the clinical stages of pancreatic cancer using GEPIA database. Results indicated that the expression levels of OAS family members were significantly different in various clinical stages (P < 0.05) (**Figure 7A**), it is obvious that the expression trend was consistent with that of overall survival curves.

The potential prognosis values of OAS1, OAS2, OAS3, and OASL in pancreatic cancer were investigated using Kaplan-Meier Plotter, GEPIA, and OncoLnc databases. Results derived from these three databases all indicated that elevated levels of OAS1, OAS2, OAS3, and OASL were associated with poor overall survival (OS) in pancreatic cancer (**Figure 7B**). Thus, the four members of OAS family might serve as prognostic indicators and even therapeutic targets for pancreatic cancer.

### Relationship Between OAS Expression and Immune Cell Infiltration

**Figure 8:**
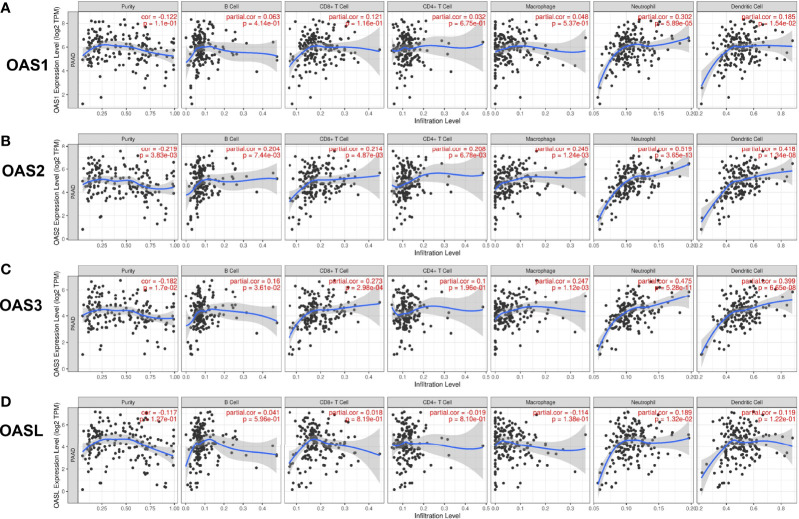
Correlation of OAS gene family with tumor immune cell infiltration in pancreatic cancer from TIMER database. **(A)** OAS1 expression had positive correlation with the infiltration level of neutrophils and dendritic cells. **(B)** OAS2 expression showed positive correlation with the infiltration levels of B cells, CD8+ T cells, CD4+ T cells, macrophage, neutrophils and dendritic cells. **(C)** OAS3 expression exhibited positive correlation with the infiltration levels of B cells, CD8+ T cells, macrophage, neutrophils and dendritic cells. **(D)** OASL expression had positive correlation with the infiltration level of neutrophils.

Tumor microenvironment (TME) has important impact on tumor growth, metabolism, metastasis, prognosis, and therapeutic response to anticancer drugs (20). We investigated the relationship between OAS family expression and immune cell infiltration in pancreatic cancer using TIMER database and results were shown in **Figure 8**. The expressions of OAS2 and OAS3 had significant negative correlations with tumor purity in pancreatic cancer (cor = −0.219 and −0.182, respectively, P < 0.05). OAS1 expression showed positive correlation with the infiltration level of neutrophils and dendritic cells (partial.cor = 0.302 and 0.185, respectively, P < 0.05) (**Figure 8A**). OAS2 expression showed positive correlation with the infiltration level of B cells, CD8+ T cells, CD4+ T cells, macrophage, neutrophils and dendritic cells (partial.cor = 0.204, 0.214, 0.208, 0.245, 0.519, 0.418, respectively, P < 0.05) (**Figure 8B**), OAS3 expression showed positive correlation with the infiltration level of B cells, CD8+ T cells, macrophage, neutrophils and dendritic cells (partial.cor = 0.16, 0.273, 0.247, 0.475, 0.399, respectively, P < 0.05) (**Figure 8C**), and OASL expression showed positive correlation with the infiltration level of neutrophils (partial.cor = 0.189, P < 0.05) (**Figure 8D**). Generally, among these immune cells, neutrophils and dendritic cells were most closely related to the occurrence and development of pancreatic cancer.

### Correlations Among OAS Family Members and Their Co-Expressed Genes in Pancreatic Cancer

**Figure 9:**
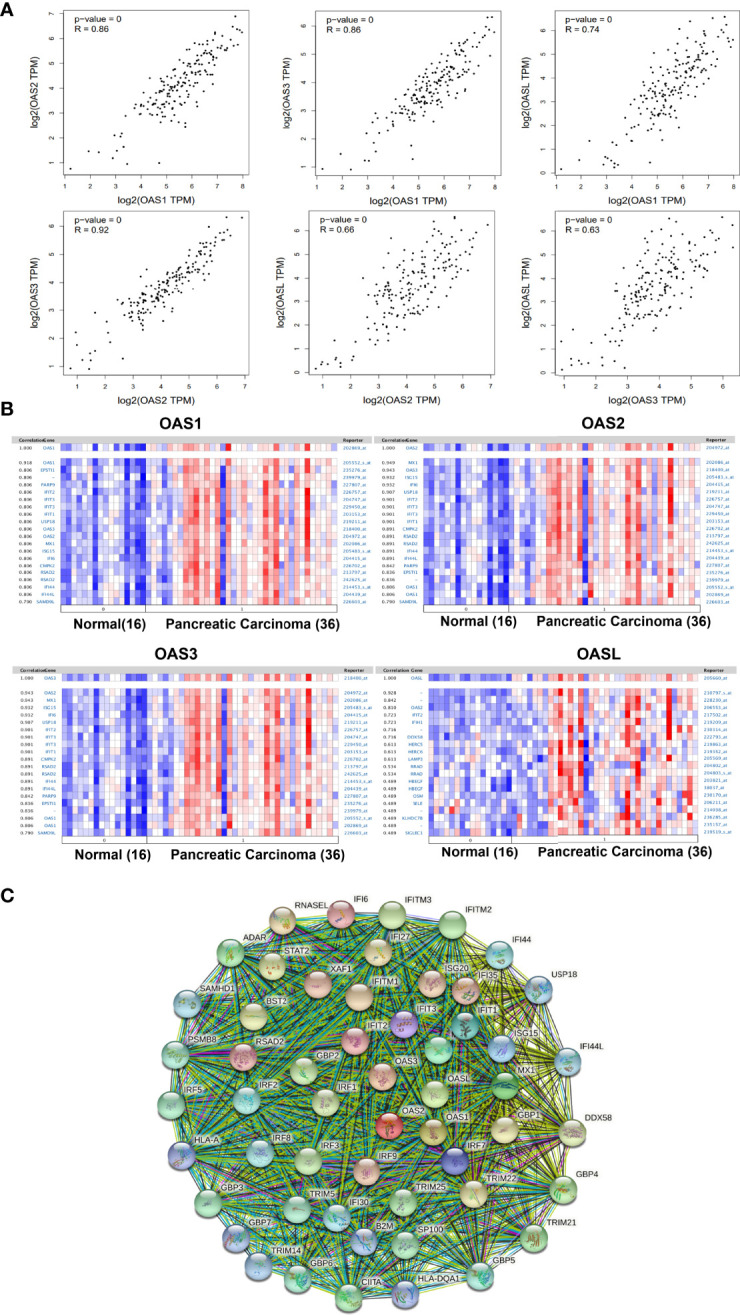
Correlations among OAS family members and co-expressed genes of OAS family in pancreatic cancer. **(A)** Correlations among OAS1, OAS2, OAS3, and OASL in GEPIA database. **(B)** Co-expressed genes relevant to OAS1, OSA2, OAS3, and OASL, respectively. **(C)** Common network for OAS gene family and their neighboring top 50 co-expression genes.

During the occurrence and development of diseases including cancers, many genes may act cooperatively or synergistically, and revealing the cooperation of genes may help to elucidate the mechanisms of a disease. By analyzing the GEPIA database, we found that OAS1 expression level was positively correlated with OAS2, OAS3, and OASL (R = 0.86, 0.86, and 0.74, respectively, P = 0) (**Figure 9A**). The expression of OAS2 was positively correlated with OAS3 and OASL (R = 0.92 and 0.66, respectively, P = 0), and OAS3 expression was positively correlated with OASL (R = 0.63, P = 0).

The co-expressed genes that related to OAS1, OAS2, OAS3, and OASL were respectively identified in Pei Pancreas dataset of Oncomine database with 16 normal pancreatic tissues and 36 pancreatic carcinoma tissues (**Figure 9B**). Results showed that OAS1 was positively correlated with EPSTI1, PARP9, IFIT2, IFIT3, IFIT1, USP18, OAS3, OAS2, MX1, ISG15, IFI6, CMPK2, RSAD2, IFI44, IFI44L, and SAMD9L. OAS2 was positively correlated with MX1, OAS3, ISG15, IFI6, USP18, IFIT2, IFIT3, IFIT1, CMPK2, RSAD2, IFI44, IFI44L, PARP9, EPSTI1, OAS1, and SAMD9L. OAS3 was positively correlated with MX1, ISG15, IFI6, USP18, IFIT2, IFIT3, IFIT1, CMPK2, RSAD2, IFI44, IFI44L, PARP9, EPSTI1, OAS1, and SAMD9L. OASL was positively correlated with OAS2, IFIT2, IFIT1, DDX58, HERC5, HERC6, LAMP3, RRAD, HBEGF, OSM, SELE, KLHDC78, and SIGLEC1. Among these genes, IFIT1, IFIT2, IFIT3, MX1, and ISG15 appeared more frequently. These genes may be functionally co-expressed with OAS gene family and important for the occurrence and development of pancreatic cancer.

Analysis of protein interaction network by STRING showed that the top 50 co-expressed genes of OAS family included MX1, MX2, IFIT1, ISG15, IRF7 IFIT3, IFI6, IFIT2, RSAD2, IFI35, XAF1, IRF9, ISG20, IFITM1, RNASEL, IFI27, TRIM22, GBP1, BST2, STAT2, DDX58, IRF1, IFITM3, GBP2, IRF3, GBP4, IFITM2, TRIM25, TRIM21, ADAR, CIITA, SP100, GBP3, IRF5, TRIM5, GBP6, PSMB8, GBP5, IRF2, IRF8, IFI44, IFI30, SAMHD1, IFI44L, USP18, TRIM14, B2M, GBP7, HLA-DQA1, and HLA-A (**Figure 9C** and **Supplementary Table 1**). These top 50 co-expression genes were chosen to perform the following functional analysis.

### GO and KEGG Pathway Enrichment Analyses on The Biological Functions of OAS Gene Family in Pancreatic Cancer

**Figure 10:**
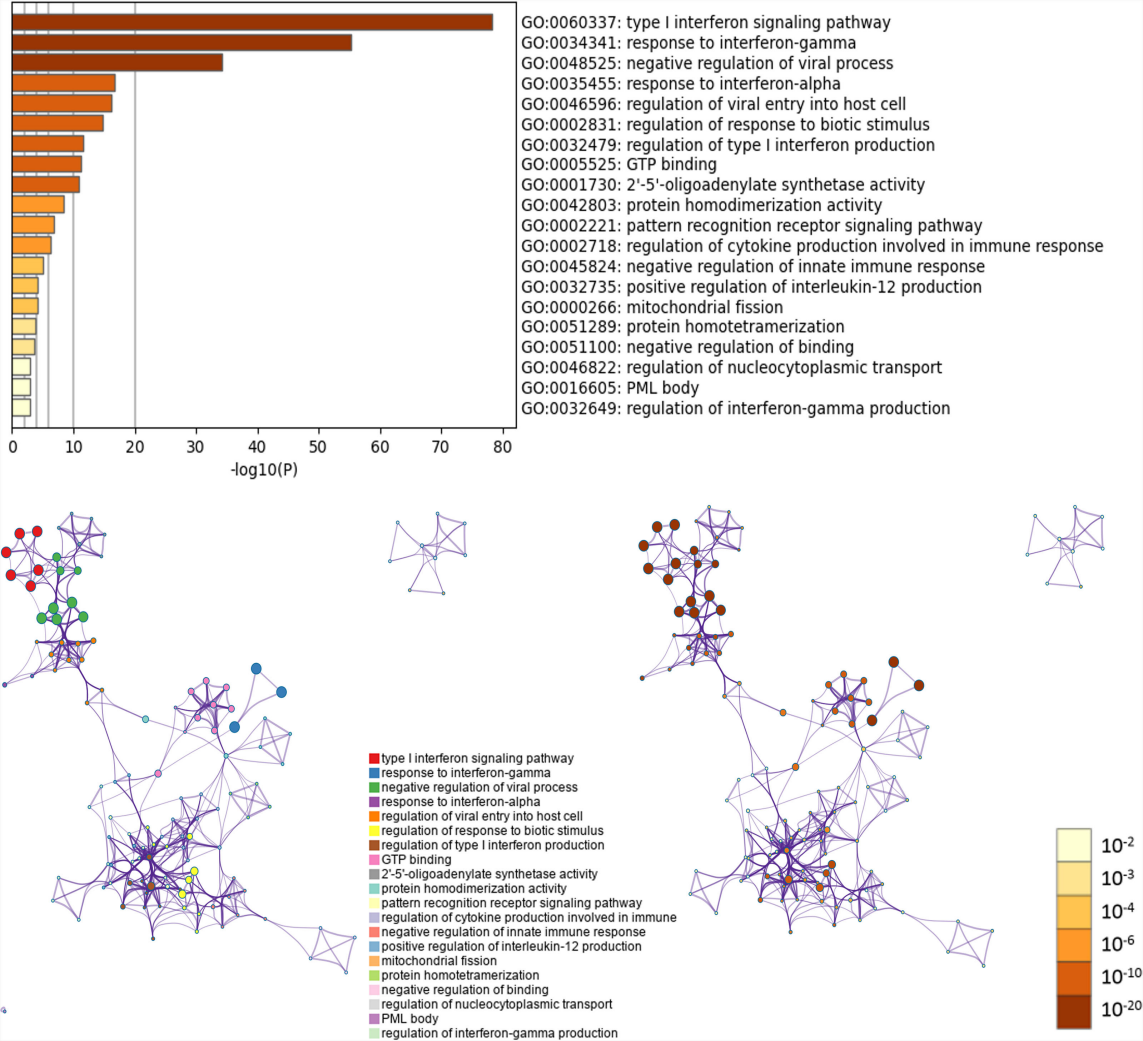
GO enrichment analysis of OAS gene family and their neighboring top 50 co-expression genes in Metascape database.

GO and KEGG pathway enrichment analyses were performed using Metascape. In GO analysis, the functional roles of genes were based on three aspects, including biological processes (BP), cellular components (CC), and molecular functions (MF). GO functional enrichment analysis revealed that target genes (OAS family and the top 50 co-expressed genes) were significantly enriched in several terms and pathways, such as GO:0060337 (BP: type I interferon signaling pathway), GO:0034341 (BP: response to interferon-gamma), GO:0048525 (BP: negative regulation of viral process), GO:0035455 (BP: response to interferon-alpha), GO:0046596 (BP: regulation of viral entry into host cell), GO:0002831 (BP: regulation of response to biotic stimulus), GO:0032479 (BP: regulation of type I interferon production), GO:0005525 (MF: GTP binding), GO:0001730 (MF: 2’-5’-oligoadenylate synthetase activity), GO:0042803 (MF: protein homodimerization activity), GO:0002221 (BP: pattern recognition receptor signaling pathway), GO:0002718 (BP: regulation of cytokine production involved in immune response), GO:0045824 (BP: negative regulation of innate immune response), GO:0032735 (BP: positive regulation of interleukin-12 production), GO:0000266 (BP: mitochondrial fission), GO:0051289 (BP: protein homotetramerization), GO:0051100 (BP: negative regulation of binding), GO:0046822 (BP: regulation of nucleocytoplasmic transport), GO:0016605 (CC: PML body), and GO:0032649 (BP: regulation of interferon-gamma production). These GO terms may play critical role in the development and progression of pancreatic cancer (**Figure 10** and **Supplementary Table 2**).

**Figure 11:**
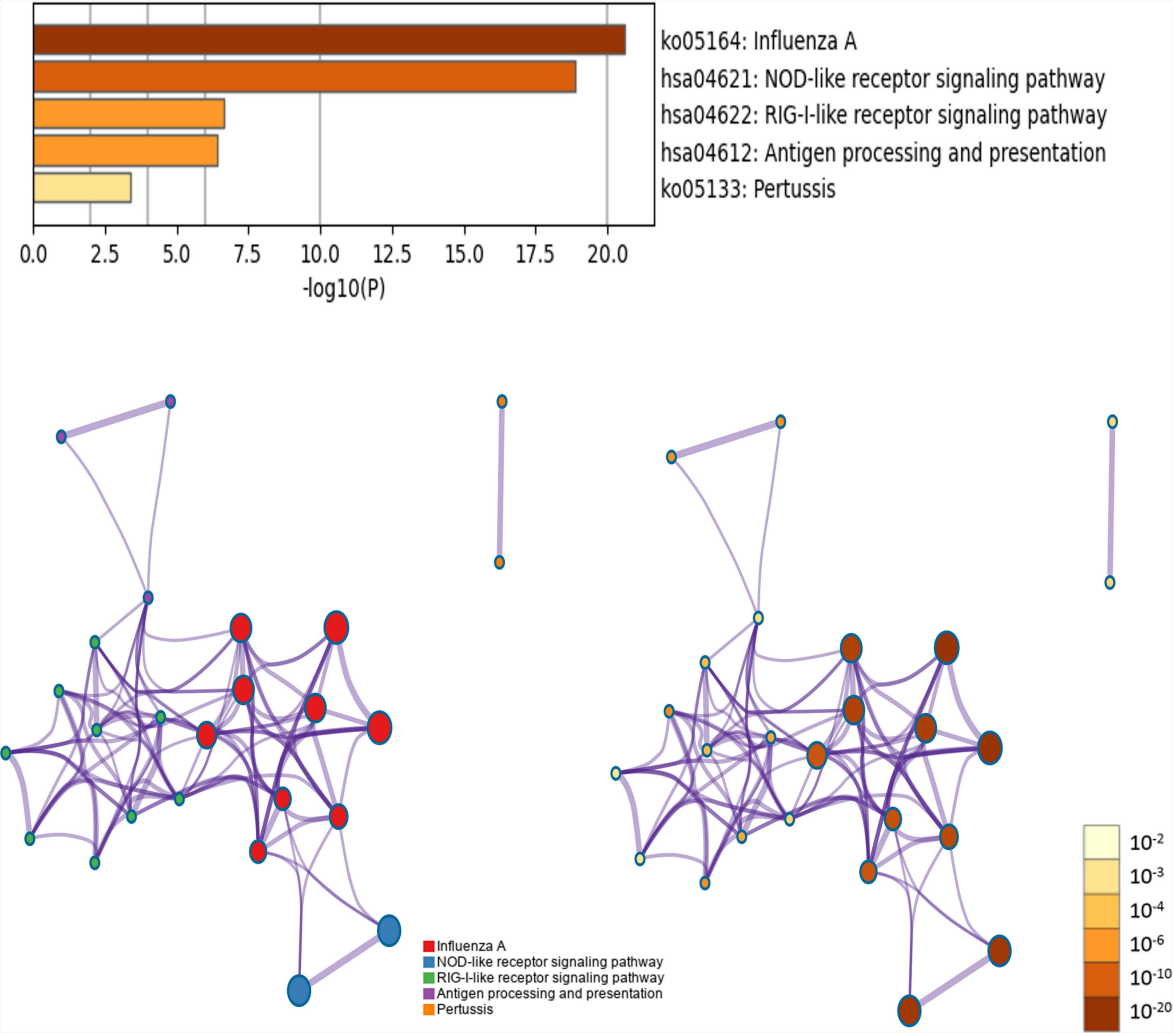
KEGG pathway enrichment analysis of OAS gene family and their neighboring top 50 co-expression genes in Metascape database.

**Figure 12:**
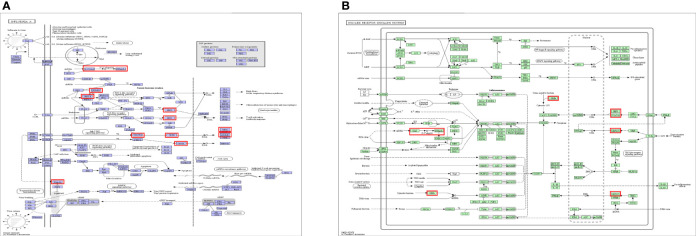
Top 2 KEGG pathways of KEGG pathway. **(A)** Ko05164: Influenza A. **(B)** hsa04621: NOD-like receptor signaling pathway.

Five KEGG pathways of target genes (OAS family and the top 50 co-expression genes) in the pathogenesis of pancreatic cancer were identified by Metascape, including ko05164 (Influenza A), hsa04621 (NOD-like receptor signaling pathway), hsa04622 (RIG-I-like receptor signaling pathway), hsa04612 (Antigen processing and presentation), and ko05133 (Pertussis) (**Figure 11** and **Supplementary Table 3**). The top two pathways were shown in **Figure 12**. Most of these functional terms and pathways were immune-related, such as GO:0034341, GO:0035455, GO:0045824, GO:0032735, GO:0032649, hsa04621, and hsa04612, indicating the correlation between immune response and tumorigenesis.

## Discussion

Pancreatic cancer is a fatal disease with poor prognosis, owing to its outbreak at late stage and pervasive therapeutic resistance (21). According to the WHO classification, the pathology of pancreatic cancer can be divided into two categories: epithelial cancer and non-epithelial cancer. Ductal adenocarcinoma originating from ductal epithelium accounts for 80−90% of pancreatic cancer (22, 23). Other types are relatively rare, including mucinous cystadenocarcinoma, acinar cell carcinoma, adenosquamous carcinoma, neuroendocrine tumors, and various mixed tumors. The increasing incidence of pancreatic cancer is thought related with lifestyles, such as smoking, alcohol, diabetes, obesity, and even has a certain genetic predisposition (24, 25). Given its unfavorable result, extensive investigations on the pathogenesis and progression of pancreatic cancer are essential to improve the survival rate.

Pancreas is an organ of poor blood supply, and pancreatic cancer is not sensitive to radiotherapy and chemotherapy, surgery is relatively the best way at present. However, the occurrence and development of pancreatic cancer is a long-term process with multiple stages and accumulation of multiple genetic changes. Moreover, the pathogenesis of pancreatic cancer is hidden, early invasion and metastasis are high, thus most patients lose the opportunity for surgery. Therefore, mechanistic exploration of the cancer is very important. Numerous studies have declared that immune cell infiltration and response to immunotherapy are determined by features of pancreatic cancer (26, 27). Under normal circumstances, the immune system can recognize and eliminate tumor cells in tumor microenvironment (TME). However, tumor cells can adopt different strategies to survive and grow, making the immune system restrained (28).

TME is quite complex in pancreatic cancer, and comprises of a variety of cell types including epithelial cancer cells, stromal cells, cancer-associated fibroblasts, immune cells, and other components of extracellular matrix that are essential to cancer progression and metastasis (29, 30). In the TME, immune cell abnormalities may promote cancer, especially, immune cell infiltration into solid tumor mass is a significant factor influencing tumor genesis and progression. However, pancreatic cancer is typically known as an immunologically ‘cold’ tumor, only a part of cancer type are immunologically active (31, 32). Pancreatic cancer presents extremely aggressive features and is associated with poor survival, this is attributed to the special features of TME, which is known to create a dense stromal formation and poorly immunogenic condition (33). The composition and abundance of immune cells in the TME are important factors that affect tumor progression and immunotherapy effect. For most pancreatic cancer patients, cytotoxic T cell infiltration is low. Although immunotherapies targeting cytotoxic T lymphocyte antigen-4 (CTLA-4), programmed cell death protein-1 (PD-1), and programmed death-ligand 1 (PD-L1) can treat several solid malignancies such as melanomas, but they are ineffective for pancreatic cancer patients (34). Therefore, it is necessary to find new immunotherapy targets for pancreatic cancer. Clinical and experimental evidence both indicate that inflammation is a significant risk factor for pancreatic cancer (35). In the present study, the expression of OAS gene family was significantly and positively correlated with the infiltration of neutrophil and dendritic cells, especially OAS2 and OAS3. OAS gene family encode innate immune proteins, playing a pivotal role in promoting sterile inflammation by regulating immune responses. Therefore, we speculate that the OAS gene family may be an important mediator linking immune cells and cancer.

Neutrophil is one of the most abundant immune cell types in pancreatic cancer environment, and is associated with a poor clinical prognosis (36). Neutrophils are produced in bone marrow from hematopoietic progenitor cells and recruited to tumors by tumor cell-derived cytokines and chemokines to participate in tumor growth. Previously, it was thought that neutrophils had little effect on cancer because of their short lifespan. However, their important role in tumor cells is increasingly recognized, because the labeling technology for studying neutrophils has been greatly developed (37). A study by Antonio et al. (38) showed that neutrophils have a shorter lifespan in wounds, but have a longer lifespan in tumors. Longer lifespan allows more time for neutrophils to act in tumor development. In the tumor metastatic process, neutrophils are responsible for the establishment of a hospitable channel that promotes the growth of disseminated pancreatic cancer cells (39, 40), and they support the survival of circulating tumor cells and promote the establishment of metastatic lesions (41, 42). Inhibiting neutrophil function may help to pancreatic cancer treatment. Nielsen et al. (36) demonstrate that lorlatinib indirectly inhibit the growth of pancreatic cancer at the primary and metastatic sites by suppressing neutrophil development in the bone marrow and modulating tumor-associated neutrophil functions in TME, providing an important basis for the immunotherapy of pancreatic cancer. Dendritic cells (DCs) are most powerful antigen-presenting cells, they are the center of immune responses, linking innate and adaptive immunity. DCs can recognize and degrade macromolecular proteins, then present them on the cell surface and deliver them to naive T cells, initiating and modulating adaptive immunity. DCs are import part of the tumor microenvironment, their particular properties can be used to fight against cancer (43, 44). Pancreatic cancer is characterized by reduced number and function of DCs which perform antigen presentation and lead to immune tolerance (45). We found that high expressions of OAS2 and OAS3 were positively correlated with the degree of infiltration of DCs in pancreatic cancer, but this result was inconsistent with the effect of DCs on cancer, the reason may be related to aberrant antigen presentation, but the specific mechanism in pancreatic cancer needs further experimental validation. DCs are broadly classified as classical (or conventional) DCs (cDCs) and plasmacytoid DCs (pDCs) (46). The antitumor process was more relevant to cDCs which transport tumor antigens to draining lymph nodes and cross-present antigens to activate cytotoxic T lymphocytes, and thus activate immunity against tumor (47). Function of pDCs was to produce significant quantities of type I interferon in response to single-stranded viral RNA and DNA (48). pDCs can assist cDCs to release IFN-α and perform antigen presentation to fight cancer, so it can also be considered as a therapeutic target (49). These advantages of DCs can be used to better fight cancer. In the present study, type I interferon signaling pathway is one result of GO enrichment analysis, its function has an important relationship with the function of pDCs. Choi et al. (50) reported that activation of type I interferon is a novel approach to activate the immune system against cancer. NOD-like receptor (NLRs) signaling pathway is another important signaling pathway that is worthy of attention. Proteins of NLRs family are a group of pattern recognition receptors (PRRs) known to mediate the initial innate immune response to cellular injury and stress (51), and they have been established as crucial regulators in inflammation-associated tumorigenesis, angiogenesis, cancer cell stemness and chemoresistance (52). NLRP3 inflammasome belongs to the family of NLRs and is the most well characterized NLRs. NLRP3 is a tripartite molecule of the nucleotide-binding domain and leucine-rich repeat family (53), Increased evidences indicate that hyperactivation of NLRP3 inflammasome is involved in a range of inflammatory diseases (54). Inflammation is an important hallmark of cancer that substantially contributes to the development and progression of malignancies (55, 56). Targeting NLRP3 or downstream signaling molecules, such as caspase-1, IL-1β or IL-18, has the potential for therapeutic benefit (57). Zhang et al. (58) revealed that 3,4-methylenedioxy-β-nitrostyrene (MNS), as a specific NLRP3 inflammasome inhibitor, could significantly decrease the migration, invasiveness, and proliferation of pancreatic cancer cells. Hao et al. (59) reported that lncRNA XLOC_000647, as the upstream regulatory non-coding RNA of NLPR3, was downregulated in pancreatic cancer and caused high expression of NLPR3, thus promoting cell proliferation, invasion, and tumor growth in pancreatic cancer. Above studies all prove that NLPR3 may potentially be a novel therapeutic target in pancreatic cancer.

There is still not much research on the role of OAS gene family in cancer. In our study, **Figure 1B** showed that OAS gene family was associated with breast cancers, head and neck cancer, liver cancer, and pancreatic cancer. However, there are only some reports on breast cancer. Zhang et al. (60) reported that OAS gene family are upregulated in breast cancer, and high mRNA expressions of OAS1 and OAS3 are correlated with poor prognosis, whereas OAS2 is associated with favorable prognosis in breast cancer. We found here that the four OAS family members were not only highly expressed, but were also closely associated with poor prognosis in pancreatic cancer. This is an important discovery. In addition, we found that all the OAS family members were significantly increased in stages IIA or IIB of pancreatic cancer. Stage II is an important transition stage, in which tumor cells begin to gradually metastasize (61). Throughout the clinical stages, the trend of these four OAS gene family members were relatively consistent, which may be related to their collaborative work. However, the relationship between such change in staging and poor prognosis still needs further investigation.

The highlight of the present study was the discovery of the relationship between OAS gene family expression and pancreatic cancer. We first found that OAS gene family was highly expressed in pancreatic cancer and caused unfavorable prognosis. As immune-related genes, members of OAS gene family may work together in the occurrence and development of pancreatic cancer and may play a promoting role by participating in the construction of TME. Currently, there are not many studies on OAS gene family in pancreatic cancer. Zhang et al. (62) found that OAS1 and OASL are prognostic genes of pancreatic adenocarcinoma by constructed RNA-binding protein-related prognostic model based on TCGA and GTEx databases. This study may assist clinicians to choose targets for immunotherapy and make personalized treatment strategy for pancreatic cancer. Tang et al. (63) reported that OAS1 and OAS3 are key gene changes in pancreatic cancer cells (BXPC-3) compared with primary pancreatic stellate cells using bioinformatics analysis. Glaß et al. (64) showed that OASL is a driver and therapeutic target candidate in pancreatic ductal adenocarcinoma. While most of the above studies are bioinformatic analyses, little experimental verification has been performed on OAS gene family in pancreatic cancer. Our present findings in both bioinformatic analyses and cellular experiments were consistent with above reports, and may provide a certain basis for future experimental studies of pancreatic cancer.

Our study has certain limitations, including lack of clinical sample validation, and deep mechanistic study, such as observing the effect of altering the effect of the OAS family gene(s) expression in pancreatic cancer cells by invasion and colony forming assays, and manipulating the OAS family gene(s) in pancreatic cancer cells on their pro- and anti-inflammatory cytokine release profile, due to campus lockdown in the COVID-19 epidemic season. However, the most valuable finding of this study was that we first found and emphasized the importance of OAS gene family in pancreatic cancer, which might become a focus in future studies of pancreatic cancer.

In conclusion, we proved high expression of OAS gene family in pancreatic cancer through analyses on a large number of public databases and validation in pancreatic cancer cell lines. We further revealed that high expression of OAS gene family was regulated by 193 TFs and 140 miRNAs. We also demonstrated that high expression of OAS gene family was related to certain clinical stage and poor prognosis of pancreatic cancer, and the function and mechanism of OAS gene family in the development of pancreatic cancer was closely related to the immune microenvironment.

For future perspective, the findings of the present study suggest that members of the OAS gene family are important in the pathogenesis and development of pancreatic cancer and may serve as biomarkers of the tumor. Targeting OAS gene family may have clinical prospects in the treatment and prevention of pancreatic cancer.

## Materials and Methods

### GTEx Analysis

The Genotype-Tissue Expression (GTEx) project (65) (https://www.gtexportal.org/home/) was established to characterize genetic effects on the transcriptome across human tissues and to link these regulatory mechanisms to trait and disease associations. At present, the project has examined 15,201 RNA-sequencing samples from 49 tissues of 838 postmortem donors. Using this database, we analyzed the expression levels of OAS gene family in various normal tissues and organs.

### ONCOMINE Dataset Analysis

Oncomine gene expression array dataset (66) (https://www.oncomine.org/resource/login.html) was used to analyze the mRNA transcriptional levels of OAS family in different cancers, and to assess the concrete expression of OAS gene family in Pei pancreas dataset and Badea pancreas dataset. In Oncomine database, the cutoffs of *P* value and fold change were defined as 0.0001 and 2, respectively.

### GEPIA Dataset Analysis

Gene Expression Profiling Interactive Analysis (GEPIA) (67) (http://gepia.cancer-pku.cn/) is an online database that facilitates the standardized analysis of RNA sequencing data from 9,736 tumor samples and 8,587 normal samples in the TCGA and GTEx datasets. In our study, GEPIA was mainly used to verify the mRNA expression level, the relationship between gene expression and cancer clinical stages, co-expressed genes, and the prognostic value of OAS family in pancreatic cancer.

### Cell Culture and Treatment

Pancreatic cancer cells lines (BxPC-3, PANC-1, CFPAC-1) and pancreatic normal cell line (hTERT-HPNE) were purchased from Shanghai Institutes for Biological Sciences, Chinese Academy of Sciences (Shanghai, China). Cells were seeded in 6-well plates at a density of 1×10^6^ cells/well. PANC-1 and hTERT-HPNE cells were cultured in DMEM supplemented with 10% fetal bovine serum and 1% penicillin and streptomycin. BxPC-3 cells were cultured with RPMI 1640 supplemented with 10% fetal bovine serum and 1% penicillin and streptomycin. CFPAC-1 cells were cultured with IMDM supplemented with 10% fetal bovine serum and 1% penicillin and streptomycin. All cells were cultured at 37°C in a humidified 5% CO_2_ atmosphere until the cell confluency reached 60% − 70%.

### RNA Isolation and Quantitative Real-Time PCR (qPCR)

qPCR was performed to examine the mRNA levels of OASs in pancreatic cancer cell lines (BxPC-3, PANC-1, CFPAC-1) and pancreatic normal cell line (hTERT-HPNE). Total RNA was extracted from cells using TRIzol (Invitrogen, Carlsbad, CA) according to the manufacturer’s instruction. qPCR was performed according to the instructions of TaKaRa TB Green Premix Ex Taq II (TaKaRa, Osaka, Japan). Primer sets for selected genes were designed by Sangon Biotech Co.,Ltd (Shanghai, China). The expression data were normalized to the reference glyceraldehyde-3-phosphate dehydrogenase (GAPDH) and the mRNA levels were calculated using the 2^−ΔΔCt^ method. Primer sequences for qPCR were as follows: GAPDH forward: 5’-CTGGGCTACACTGAGCACC-3’, GAPDH reverse: 5’-AAGTGGTCGTTGAGGGCAATG-3’. OAS1 forward: 5’-AGTTGACTGGCGGCTATAAAC-3’, OAS1 reverse: 5’-GTGCTTGACTAGGCGGATGAG-3’. OAS2 forward: 5’-AGGTGGCTCCTATGGACGG-3’, OAS2 reverse: 5’-TTTATCGAGGATGTCACGTTGG-3’. OAS3 forward: 5’- GAAGGAGTTCGTAGAGAAGGCG -3’, OAS3 reverse: 5’-CCCTTGACAGTTTTCAGCACC-3’. OASL forward: 5’-CCCTTGACAGTTTTCAGCACC-3’, OASL reverse: 5’-CTTCAGCTTAGTTGGCCGATG-3’.

### Western Blotting

Total proteins for western blotting were extracted from pancreatic cancer cell lines (BxPC-3, PANC-1, CFPAC-1) and pancreatic normal cell line (hTERT-HPNE). The protein concentration in all samples was determined using the bicinchoninic acid (BCA) assay (Solarbio Co., Ltd, Beijing, China). A total amount of 40 μg extracted protein of each sample were separated by 10% SDS-PAGE. Then, proteins from the SDS-PAGE gel were transferred onto a polyvinylidene fluoride (PVDF) membrane (Millipore, Billerica, MA, USA). Membranes were blocked with 5% nonfat milk for 2−3 h at 20−25°C. The membranes were then incubated with the primary antibodies overnight at 4 °C respectively. The membrane was washed with TBST and then incubated with the secondary antibody conjugated with horseradish peroxidase for 2 h at 20−25 °C. The ECL reagent (Millipore, Billerica, MA, USA) was added and the blots were scanned using ChemiDoc™ XRS (Bio-Rad Laboratories, Hercules, CA, USA). The gray values of protein bands were determined using Image Lab 2.0 (Genmall Biotechnology Co.,Ltd, Wuhan, China) and β-actin (ZSGB-Bio, China) was used for normalization. The primary antibodies (anti-OAS1, anti-OAS2, anti-OAS3) were purchased from Peprotech (New Jersey, USA), anti-OASL was purchased from Abcam (Cambridge, MA, USA), the secondary antibodies were purchased from Zhongshan Golden bridge Biotechnology (Beijing, China).

### The Human Protein Atlas Project Analysis

The Human Protein Atlas Project (HPA) (68) (http://www.proteinatlas.org/) is a free public database that provides information about human encoded proteins. It is dedicated to the mRNA and protein expression information of all 24,000 genes encoding human proteins in 44 normal tissues, 18 tumor tissues, 69 cell lines and 18 blood cell lines. The immunohistochemistry images detecting protein expressions of OAS gene family in pancreatic cancer and normal pancreatic tissue were retrieved from HPA.

### TF-Target and miRNA-Target of OAS Gene Family

Transcription factors (TFs) are key regulators that modulate the expression of target genes by recognizing specific DNA sequences to control chromatin and transcription, forming a complex system that guides expression of the genome (69). hTFtarget (70) (http://bioinfo.life.hust.edu.cn/hTFtarget) provides comprehensive TF-target regulations from large-scale of ChIP-Seq data of human TFs in 569 conditions. miRNAs are non-coding RNA molecules which serve a crucial role in regulating a spectrum of basic cellular processes and they may induce RNA-silencing and work as post-DNA transcription regulators. In this work, we used Starbase (71) (http://starbase.sysu.edu.cn/) and Targetscan (72) (http://www.targetscan.org/vert_72/) to locate the upstream miRNAs.

### Kaplan-Meier Plotting and OncoLnc Analysis

The Kaplan-Meier (KM) plotter (73) (https://kmplot.com/analysis/) is committed to analyze the survival biomarkers across 21 cancer types, based on sources including Gene Expression Omnibus database (GEO), European Genome-phenome Archive (EGA), and the Cancer Genome Atlas (TCGA). One of prognostic values of the OAS family for overall survival (OS) were calculated using the KM plotter.

OncoLnc (74) (http://www.oncolnc.org/) is an useful online tool for downloading clinical data coupled to expression data, and exploring survival correlations for genes. It contains survival data for 8,647 patients from 21 cancer studies based on TCGA, which generate high quality OS plots for further analyses in this study.

### TIMER Analysis

Tumor Immune Estimation Resource (TIMER) (75) (https://cistrome.shinyapps.io/timer/) is a database designed for analyzing immune cell infiltrates in multiple cancers based on 32 cancer types and 10,897 samples from TCGA. Using this database, we estimated tumor immune infiltration by B cells, CD4+ T cells, CD8+ T cells, neutrophils, macrophages, and dendritic cells. *P* < 0.05 was considered statistically significant.

### Interactions of OAS Gene Family by STRING

STRING (76) (https://string-db.org/) is an online database developed by European Molecular Biology Laboratory on functional association for genes. It includes 5,090 species, more than 20 million proteins and 3 billion interactions. In this study, top 50 neighbor genes related to OAS gene family were collected and integrated analysis was performed *via* STRING.

### Gene Ontology and Kyoto Encyclopedia of Genes and Genomes Pathway Enrichment Analysis by Metascape

Gene Ontology (GO) is a standardized classification system of gene function which is used to comprehensively describe the properties of genes. In GO enrichment analysis, biological processes (BP), cellular components (CC), and molecular functions (MF) are included. Kyoto Encyclopedia of Genes and Genomes (KEGG) is a system used to comprehensively analyze the genomic information, gene function and relationship with targets of pathways.

Metascape (77) (http://metascape.org) is a web-based portal designed to provide a comprehensive gene list annotation and analysis resource for experimental biologists. It combines functional enrichment, interactome analysis, gene annotation, and membership search to leverage over 40 independent knowledgebases within one integrated portal. In our study, Metascape was used to analyze GO and KEGG pathway enrichment, with P value < 0.01, minimum overlap = 3 and enrichment factor > 1.5 as the criteria.

### Statistical Analysis

All statistical analyses were performed using GraphPad Prism 5.0 software. Data were presented as mean ± standard deviation (SD). One-way ANOVA was used to compare the means of sample groups. Statistical significance was set at P < 0.05.

## Data Availability Statement

The original contributions presented in the study are included in the article/**Supplementary Material**. Further inquiries can be directed to the corresponding authors.

## Author Contributions

L-JG carried out experiments, data analysis and drafted manuscript. J-LL performed parts of experiments and data analysis. R-RY, Z-MH, MY, CX, helped drew some figures. L-JG and J-MC supervised the study and revised manuscript. All authors contributed to the work and approved the submission.

## Funding

This study was supported by Key Medical Science and Technology Program of Shanxi Province (2020XM01), and Shanxi “1331” Project Quality and Efficiency Improvement Plan (1331KFC), Basic Research Program of Shanxi Province (202103021223238).

## Conflict of Interest

The authors declare that the research was conducted in the absence of any commercial or financial relationships that could be construed as a potential conflict of interest.

## Publisher’s Note

All claims expressed in this article are solely those of the authors and do not necessarily represent those of their affiliated organizations, or those of the publisher, the editors and the reviewers. Any product that may be evaluated in this article, or claim that may be made by its manufacturer, is not guaranteed or endorsed by the publisher.
