# Supplementary material for: Biological Characterization and Clinical Value of OAS Gene Family in Pancreatic Cancer
Source: Front Oncol. 2022 Jun 3;12:884334. doi: 10.3389/fonc.2022.884334 (PMC9205247; doi:10.3389/fonc.2022.884334)
Supplement: Supplementary file 1 [file DataSheet_1.docx]

**Supplementary Material**

| **SUPPLEMENTARY TABLE 1. Top 50 genes most relevant to the OAS family** | | | | | | | | | |
| --- | --- | --- | --- | --- | --- | --- | --- | --- | --- |
| **Predicted Functional Partners** | **Score** | **Predicted Functional Partners** | **Score** | **Predicted Functional Partners** | **Score** | **Predicted Functional Partners** | **Score** | **Predicted Functional Partners** | **Score** |
| MX1 | 0.999 | XAF1 | 0.987 | DDX58 | 0.964 | CIITA | 0.948 | IFI44 | 0.937 |
| MX2 | 0.997 | IRF9 | 0.984 | IRF1 | 0.963 | SP100 | 0.947 | IFI30 | 0.936 |
| IFIT1 | 0.997 | ISG20 | 0.983 | IFITM3 | 0.96 | GBP3 | 0.947 | SAMHD1 | 0.931 |
| ISG15 | 0.997 | IFITM1 | 0.983 | GBP2 | 0.957 | IRF5 | 0.944 | IFI44L | 0.931 |
| IRF7 | 0.996 | RNASEL | 0.979 | IRF3 | 0.956 | TRIM5 | 0.944 | USP18 | 0.93 |
| IFIT3 | 0.995 | IFI27 | 0.978 | GBP4 | 0.953 | GBP6 | 0.94 | TRIM14 | 0.93 |
| IFI6 | 0.994 | TRIM22 | 0.975 | IFITM2 | 0.952 | PSMB8 | 0.94 | B2M | 0.93 |
| IFIT2 | 0.993 | GBP1 | 0.973 | TRIM25 | 0.951 | GBP5 | 0.939 | GBP7 | 0.928 |
| RSAD2 | 0.993 | BST2 | 0.97 | TRIM21 | 0.949 | IRF2 | 0.939 | HLA-DQA1 | 0.927 |
| IFI35 | 0.99 | STAT2 | 0.97 | ADAR | 0.949 | IRF8 | 0.938 | HLA-A | 0.926 |

**Supplementary Table**

| **SUPPLEMENTARY TABLE 2. GO analysis of top 50 genes closely related to OAS family** | | | | |
| --- | --- | --- | --- | --- |
| **Category** | **Term** | **Description** | **LogP** | **Gene Symbols** |
| BP | GO:0060337 | type I interferon signaling pathway | -78.3356 | ADAR,BST2,IFI6,GBP2,IRF8,IFI27,IFI35,IFIT2,IFIT1,IFIT3,IRF1,IRF2,IRF3,IRF5,IRF7,ISG20,MX1,MX2,OAS1,OAS2,OAS3,PSMB8,RNASEL,SP100,STAT2,IFITM1,OASL,ISG15,IRF9,IFITM3,IFITM2,USP18,SAMHD1,XAF1,RSAD2,GBP1,GBP3,TRIM25,TRIM22,IFI44L,DDX58,TRIM5,IFI44,HLA-A |
| BP | GO:0034341 | response to interferon-gamma | -55.2777 | B2M,BST2,GBP1,GBP2,GBP3,IRF8,IRF1,IRF2,IRF3,IRF5,IRF7,CIITA,OAS1,OAS2,OAS3,SP100,TRIM21,TRIM25,IFITM1,OASL,TRIM22,IRF9,IFITM3,IFI30,IFITM2,TRIM5,GBP4,GBP5,GBP6,GBP7,HLA-DQA1,HLA-A |
| BP | GO:0048525 | negative regulation of viral process | -34.1737 | BST2,IFIT1,ISG20,CIITA,MX1,OAS1,OAS2,OAS3,RNASEL,TRIM21,TRIM25,IFITM1,OASL,ISG15,TRIM14,IFITM3,IFITM2,TRIM5,RSAD2,ADAR,TRIM22,IFI27 |
| BP | GO:0035455 | response to interferon-alpha | -16.6738 | ADAR,BST2,IFIT2,IFIT3,MX2,IFITM1,IFITM3,IFITM2,IRF1,XAF1 |
| BP | GO:0046596 | regulation of viral entry into host cell | -16.2364 | CIITA,TRIM21,TRIM25,IFITM1,TRIM14,TRIM22,IFITM3,IFITM2,TRIM5,BST2,IFIT1,IRF3,DDX58,SP100,IFI27,PSMB8,IRF5,IRF8 |
| BP | GO:0002831 | regulation of response to biotic stimulus | -14.7971 | ADAR,IFI35,IFIT1,IRF1,IRF3,IRF7,PSMB8,RNASEL,TRIM21,USP18,DDX58,SAMHD1,TRIM5,GBP5,B2M,GBP1,RSAD2,IRF5,CIITA,HLA-A,HLA-DQA1 |
| BP | GO:0032479 | regulation of type I interferon production | -11.5552 | IRF8,IRF1,IRF3,IRF5,IRF7,TRIM21,TRIM25,ISG15,DDX58,B2M,BST2,GBP1,HLA-A,RSAD2,GBP5,ADAR,IFI6,IFIT1 |
| BP | GO:0002221 | pattern recognition receptor signaling pathway | -6.78846 | IFI35,IRF1,IRF3,IRF7,TRIM25,DDX58,RSAD2,B2M,IRF8,IFIT3,IFITM1,IFI30,ISG15 |
| BP | GO:0002718 | regulation of cytokine production involved in immune response | -6.22915 | B2M,BST2,DDX58,RSAD2,PSMB8,IFI30,SAMHD1,IRF1,IRF7,IFIT1,GBP1,TRIM14,IFITM3,TRIM5,HLA-DQA1,HLA-A |
| BP | GO:0045824 | negative regulation of innate immune response | -5.12533 | ADAR,HLA-A,TRIM21,SAMHD1,BST2,GBP1,IRF1,IFIT1 |
| BP | GO:0032735 | positive regulation of interleukin-12 production | -4.20333 | IRF8,IRF1,IRF5,B2M,IRF3,TRIM5 |
| BP | GO:0000266 | mitochondrial fission | -4.13932 | MX1,MX2,STAT2,IFI6,IFIT2 |
| BP | GO:0051289 | protein homotetramerization | -3.76471 | B2M,SAMHD1,GBP5 |
| BP | GO:0051100 | negative regulation of binding | -3.59454 | B2M,IFIT2,IFIT1,SP100,TRIM21 |
| BP | GO:0046822 | regulation of nucleocytoplasmic transport | -2.98751 | IFI27,MX2,SP100,IFI6,MX1,TRIM14,RSAD2 |
| BP | GO:0032649 | regulation of interferon-gamma production | -2.93974 | HLA-A,IRF8,ISG15,ADAR,B2M,IRF7 |
| CC | GO:0016605 | PML body | -2.97539 | ISG20,CIITA,SP100 |
| MF | GO:0005525 | GTP binding | -11.2398 | GBP1,GBP2,GBP3,CIITA,MX1,MX2,IFI44L,SAMHD1,GBP4,GBP5,GBP6,GBP7,B2M,IRF8,IRF3,IRF5,OAS2,ISG15,IFI44,TRIM5,IFITM3,HLA-A |
| MF | GO:0001730 | 2'-5'-oligoadenylate synthetase activity | -10.9183 | OAS1,OAS2,OAS3,OASL,ADAR,DDX58 |
| MF | GO:0042803 | protein homodimerization activity | -8.39403 | B2M,BST2,GBP1,GBP2,GBP3,IRF3,TRIM21,TRIM14,TRIM22,TRIM5,GBP4,GBP5,HLA-A,HLA-DQA1,PSMB8 |

| **SUPPLEMENTARY TABLE 3. KEGG pathway analysis of the top 50 genes closely related to OAS family** | | | |
| --- | --- | --- | --- |
| **Term** | **Description** | **LogP** | **Symbols** |
| ko05164 | Influenza A | -20.5961 | ADAR,HLA-DQA1,IRF3,IRF7,CIITA,MX1,OAS1,OAS2,OAS3,RNASEL,STAT2,TRIM25,IRF9,DDX58,RSAD2,IFIT1,IRF1,HLA-A,SP100,B2M,ISG15 |
| hsa04621 | NOD-like receptor signaling pathway | -18.8853 | GBP1,GBP2,GBP3,IRF3,IRF7,OAS1,OAS2,OAS3,RNASEL,STAT2,IRF9,GBP4,GBP5,GBP7 |
| hsa04622 | RIG-I-like receptor signaling pathway | -6.62701 | IRF3,IRF7,TRIM25,ISG15,DDX58,ADAR,SP100,IRF9,STAT2,IRF5, HLA-A |
| hsa04612 | Antigen processing and presentation | -6.41857 | B2M,HLA-A,HLA-DQA1,CIITA,IFI30,IRF3,DDX58 |
| ko05133 | Pertussis | -3.37231 | IRF8,IRF1,IRF3 |

**Supplementary Figure**


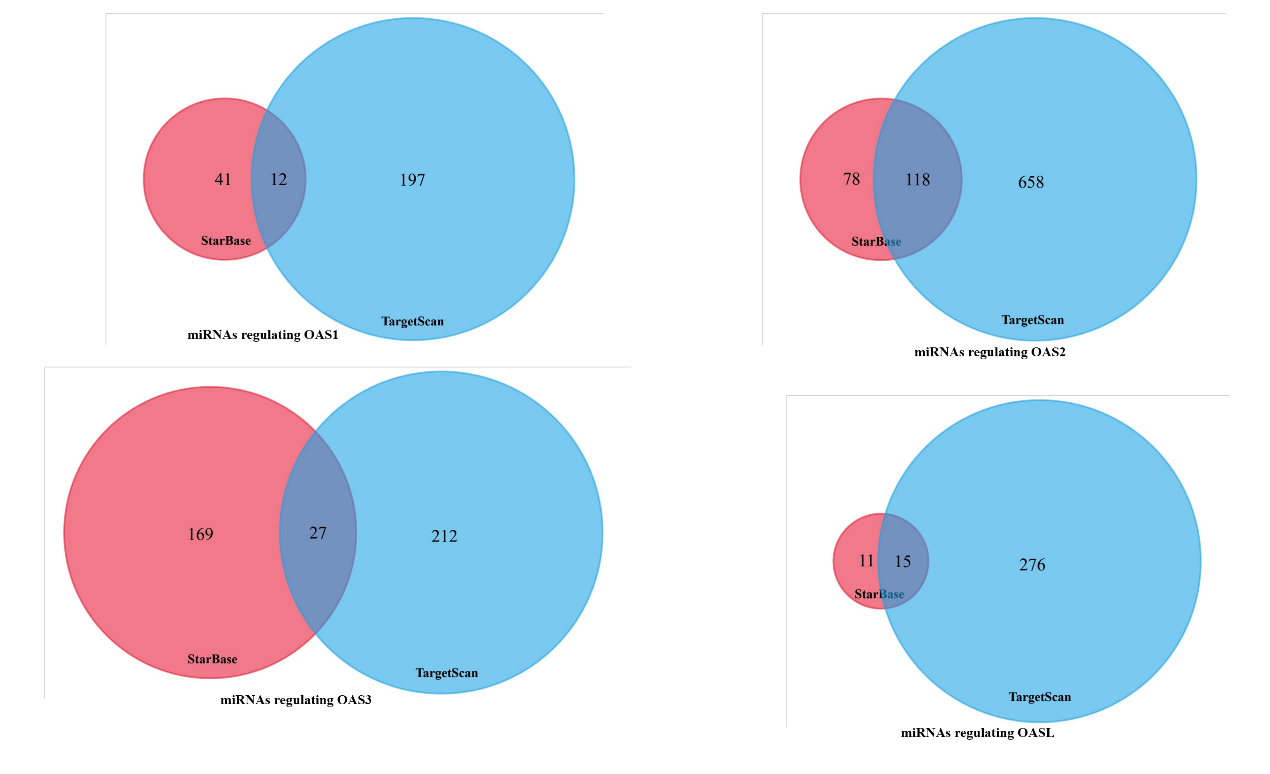


**SUPPLEMENTARY FIGURE 1.** The co-expressed miRNAs that regulate the expressions of OAS1, OAS2, OAS3, and OASL. Data were mined from Starbase and Targetscan databases.
