# Supplementary material for: Biological Characterization and Clinical Value of OAS Gene Family in Pancreatic Cancer
Source: Front Oncol. 2022 Jun 3;12:884334. doi: 10.3389/fonc.2022.884334 (PMC9205247; doi:10.3389/fonc.2022.884334)
Supplement: Supplementary file 2 [file DataSheet_2.docx]

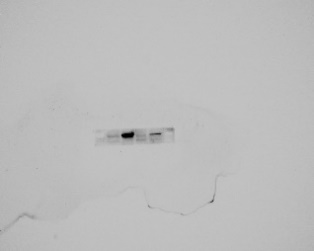

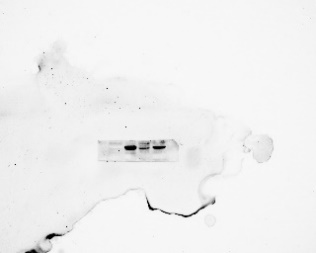


**OAS2 70KD**

**hTERT-HPNE、BXPC-3、PANC-1、CFPAC-1**

**OAS1 48KD**

**hTERT-HPNE， BXPC-3 PANC-1 CFPAC-1**


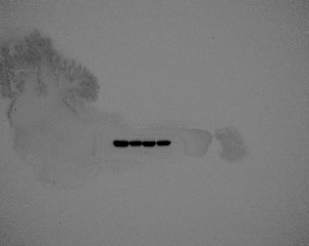

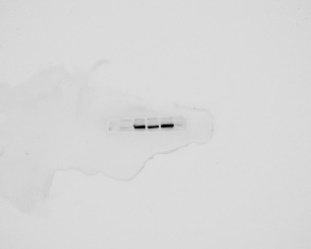


**hTERT-HPNE， BXPC-3， PANC-1， CFPAC-1**

**hTERT-HPNE， BXPC-3， PANC-1， CFPAC-1**

**β-actin 42KD**

**OAS3 120KD**


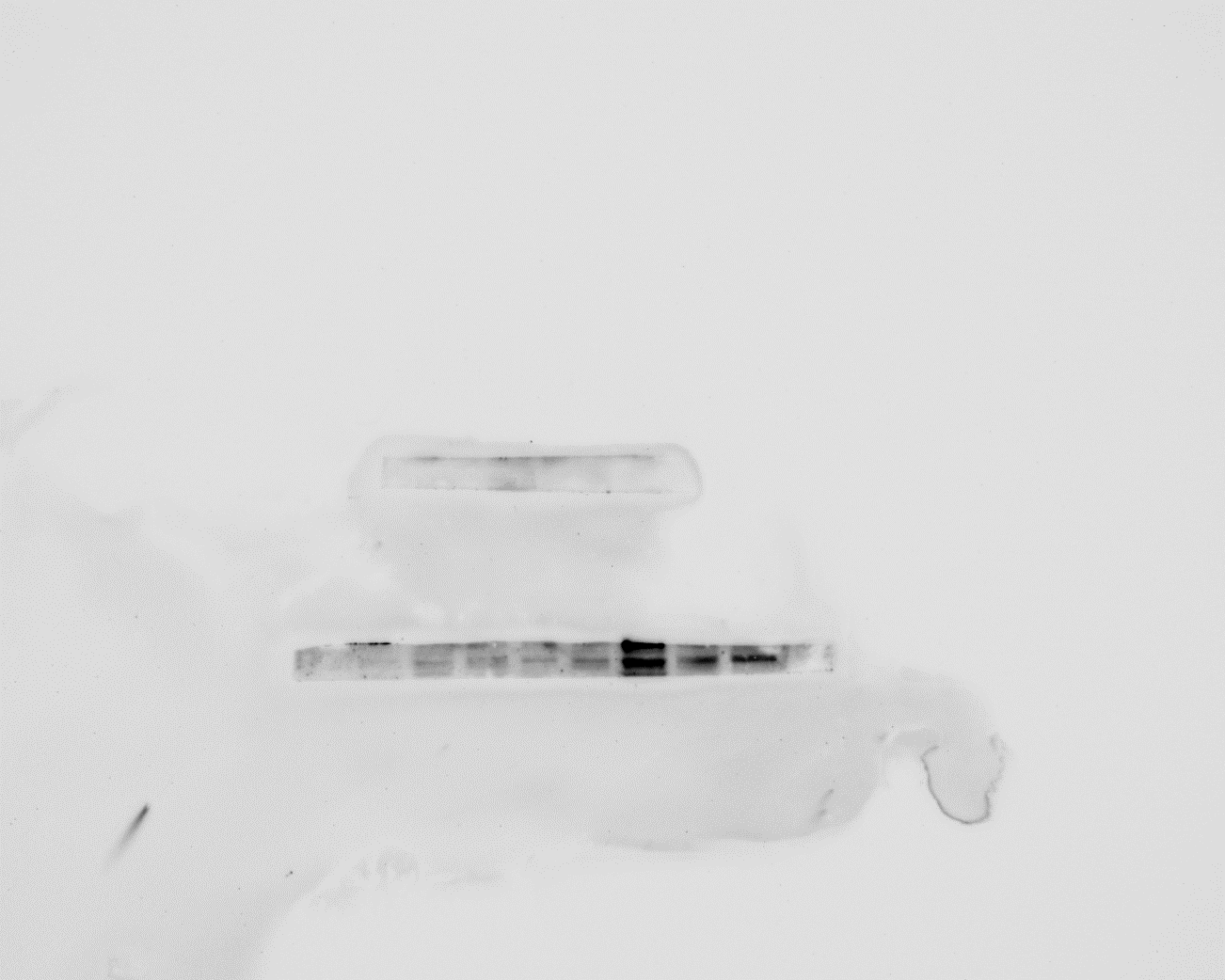

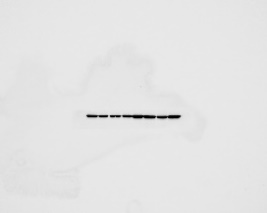


**OASL 59KD**

**hTERT-HPNE，BXPC-3，PANC-1，CFPAC-1**

**hTERT-HPNE，BXPC-3，PANC-1，CFPAC-1**

**β-actin 42KD**
